# Endovascular Management of Traumatic Intracranial Aneurysms Using the Pipeline Flex Embolization Device With Shield Technology Under Single Antiplatelet Therapy: A Case Report and Literature Review

**DOI:** 10.7759/cureus.90284

**Published:** 2025-08-17

**Authors:** Jun Isozaki, Hidetaka Onodera, Masao Shioda, Katsumi Sakata, Tetsuya Yamamoto

**Affiliations:** 1 Department of Neurosurgery, Yokohama City University Medical Center, Yokohama, JPN; 2 Department of Neurosurgery, St. Marianna University School of Medicine Hospital, Kawasaki, JPN

**Keywords:** 2-methacryloyloxyethyl phosphorylcholine polymer, dual antiplatelet therapy, flow diverter stents, minimized antiplatelet, parent vessel preservation, pipeline flex embolization device, shield technology, single antiplatelet therapy, traumatic intracranial aneurysm

## Abstract

Traumatic intracranial aneurysms (TICAs) are rare but critical lesions that develop following head injury, carrying a high risk of rupture due to their nature as pseudoaneurysms. Traditional treatments, such as surgical clipping/ligation and coil embolization, have been used to manage TICAs; however, these approaches have significant limitations, including high invasiveness and risk of rebleeding. Flow diverter stents (FDS) have emerged as a promising parent vessel-preserving option, yet managing periprocedural antiplatelet therapy remains a significant challenge, particularly in polytrauma patients. A critical question persists: can FDS be safely and effectively deployed with a minimized antiplatelet burden in patients at high risk of bleeding? This report describes a single case and reviews existing literature on FDS treatment for TICAs, specifically highlighting reports demonstrating the safety and efficacy of single antiplatelet therapy (SAPT) using the Pipeline Flex Embolization Device with Shield Technology (PED-Shield, Medtronic Inc., Irvine, CA, USA). The patient was a woman who sustained a severe head injury from traffic trauma, presenting with bilateral petrous bone fractures, a clivus fracture, and subarachnoid hemorrhage within the basal cisterns and bilateral Sylvian fissures. Initial CT angiography (CTA) on the day of injury showed no aneurysm; however, CTA on post-injury day 8 revealed a new pseudoaneurysm at the petrous-to-cavernous junction of the right internal carotid artery. Given her high bleeding risk due to polytrauma and a brain contusion necessitating decompressive craniectomy on post-injury day 2, PED-Shield was deployed under SAPT, with aspirin as the sole antiplatelet agent. The postoperative course was uneventful. Although the patient experienced attention deficits due to the cerebral contusion, she is living independently with a modified Rankin Scale score of 3. Angiography at 25 days confirmed complete aneurysm occlusion with excellent parent vessel preservation; this favorable outcome persisted at the seven-month follow-up. Our experience suggests that FDS can effectively prevent early rupture and achieve successful occlusion in TICAs. This observation aligns with existing literature demonstrating high complete occlusion rates and the ability to avoid delayed rupture with FDS treatment of TICAs. Furthermore, this case highlights a particularly significant advancement: despite no previous reports of PED-Shield use under SAPT in trauma cases, its antithrombogenic surface coating allowed treatment with SAPT, effectively mitigating the constraints of dual antiplatelet therapy (DAPT) in polytrauma patients. This approach is supported by several reports indicating the safety and efficacy of minimized antiplatelet therapy with PED-Shield in nontraumatic cases. FDS can preserve the parent vessel while ensuring durable aneurysm exclusion. In polytrauma patients, PED-Shield may reduce the limitations imposed by DAPT. This strategy represents a valuable addition to the treatment armamentarium for TICAs.

## Introduction

Traumatic intracranial aneurysms (TICAs) are rare but potentially fatal lesions that develop after head injury. Most TICAs are pseudoaneurysms, inherently prone to rapid growth and rupture, with a mortality rate of up to 50% upon rupture [1,2]. This grave prognosis necessitates prompt and aggressive intervention.

Traditionally, surgical and endovascular coiling techniques have been employed, but these methods present significant challenges. Open surgery is highly invasive and carries a substantial risk of rupture, often requiring parent vessel sacrifice [3]. Coiling, while less invasive, raises concerns about rebleeding and recurrence in pseudoaneurysms due to the absence of a true vessel wall [4]. These limitations underscore the need for safer, parent vessel-preserving, and more definitive treatment options.

Flow diverter stents (FDS) have emerged as a promising endovascular solution, enabling parent vessel preservation while achieving durable aneurysm occlusion through flow diversion and vessel remodeling. FDS have demonstrated excellent outcomes in the management of complex intracranial aneurysms [5-9]. However, their use in acute trauma patients presents a critical dilemma: FDS typically require dual antiplatelet therapy (DAPT), which poses a significant hemorrhagic risk in polytrauma patients.

A central challenge in this context is determining whether FDS can be safely and effectively deployed in high-risk trauma patients with a minimized antiplatelet regimen, thereby mitigating DAPT-related bleeding risks while still ensuring long-term parent vessel preservation and aneurysm cure.

This report describes a case of TICA successfully treated with the Pipeline Flex Embolization Device with Shield Technology (PED-Shield, Medtronic Inc., Irvine, CA, USA), a flow diverter with an antithrombogenic surface coating, under single antiplatelet therapy (SAPT). To our knowledge, this represents the first documented application of this strategy in a traumatic setting. Our experience suggests that this approach provides a crucial balance between effective aneurysm exclusion and minimized hemorrhagic risk, potentially expanding the therapeutic armamentarium for these challenging lesions.

## Case presentation

**Figure 1:**
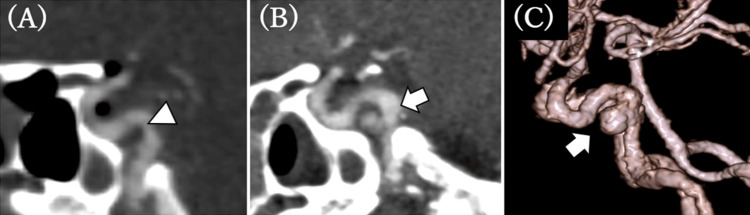
Comparison of CTA images from Day 0 (A) and Day 8 (B, C) (A) Initial CTA on Day 0 shows no evidence of an aneurysm (arrowhead). (B, C) Follow-up CTA on Day 8 demonstrates a newly developed aneurysm measuring 5 mm at its maximum diameter (arrow). CTA, CT angiography

A 46-year-old woman was struck by a 2-ton truck while cycling at approximately 30 km/h. On arrival, her Glasgow Coma Scale score was E1V1M4, indicating severe disturbance of consciousness. Initial head CT and CT angiography (CTA) revealed bilateral longitudinal fractures of the petrous bones crossing the carotid canals, an upper clival transverse fracture, thin subarachnoid hemorrhage in the basal cisterns and bilateral Sylvian fissures, and intracranial air. No aneurysm or vascular injury was identified (Figure 1). On post-injury day 1, the patient’s right frontal contusion worsened, causing right uncal herniation, and she underwent right decompressive craniectomy with intracranial pressure sensor placement. On post-injury day 8, CTA demonstrated a new 5 mm aneurysm at the petrous-to-cavernous junction of the right internal carotid artery (ICA), consistent with a traumatic pseudoaneurysm, which had been absent on the initial imaging.

Given the high rupture risk of the pseudoaneurysm and the difficulty of accessing it via open surgery, we determined that standalone FDS placement would best avoid intrasaccular manipulation while preserving the parent vessel. We selected the PED-Shield, which features an antithrombogenic surface coating, and opted for SAPT rather than DAPT because the procedure was performed in the subacute phase after decompressive craniectomy, and additional tibial fracture surgery was planned. The patient and her family received a detailed explanation of the procedure, management, and associated risks, and written informed consent was obtained prior to surgery.

**Figure 2:**
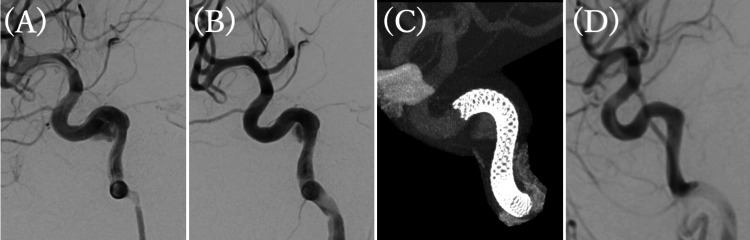
Images during treatment (A) Lateral view of DSA prior to FDS deployment, demonstrating an increase in aneurysm size compared with prior imaging. (B, C) Lateral views of DSA and cone-beam CT after FDS deployment. DSA shows an eclipse sign within the aneurysm, and the FDS extends from the cavernous to the petrous portion, covering the aneurysm. (D) Lateral view of DSA two months post-injury, showing no opacification of the aneurysm, consistent with complete occlusion. DSA, digital subtraction angiography; FDS, flow diverter stents

On post-injury day 11, the patient received a 300 mg loading dose of aspirin immediately before treatment. Heparin was administered with monitoring and adjustment of the activated clotting time during the procedure. Under general anesthesia, a 6 Fr sheath was placed in the right femoral artery, and a 6 Fr Optimo (Tokai Medical Products, Inc., Kasugai, Japan) was navigated into the right ICA. Angiography revealed a fusiform enlargement with an indistinct neck, which appeared larger than on prior CTA. A Phenom 27 microcatheter (Medtronic Inc.) was advanced through the cavernous segment, and a PED-Shield (3.75 mm × 25 mm) was deployed from the cavernous portion to the petrous segment, landing on normal vessel walls at both ends. Although some intra-aneurysmal flow persisted, the appearance of an eclipse sign confirmed adequate flow diversion, and the procedure was concluded (Figure 2).

The patient experienced no periprocedural complications. Cerebral angiography was performed on post-injury days 18 and 25. On day 25, complete aneurysm occlusion with preservation of the parent vessel was confirmed. She subsequently underwent tibial fracture fixation and cranioplasty and was transferred for rehabilitation with a modified Rankin Scale score of 3. Follow-up angiography at two months post-injury demonstrated sustained complete occlusion of the aneurysm and preservation of the parent vessel. Additionally, an MRI performed at seven months showed a favorable course with no recurrence.

## Discussion

TICAs account for only 0.15-0.40% of all intracranial aneurysms [10]. They result from direct vessel injury caused by blunt or penetrating head trauma or from shear forces due to rotational acceleration, and approximately 80% are pseudoaneurysms [11,12]. Pseudoaneurysms undergo rapid morphological changes and growth, with the highest risk of rupture occurring two to three weeks after injury, carrying a mortality rate of up to 50% upon rupture [1,2]. They are particularly associated with fractures of the sphenoid bone, petrous carotid canal, and occipital condyle; thus, skull base fractures involving these sites warrant aggressive vascular screening [2,13,14]. Our case, involving an ICA aneurysm in the petrous segment that was not visible on initial imaging but became evident on day 8, exemplifies the delayed formation typical of TICAs.

Historically, surgical techniques such as trapping, wrapping, carotid ligation, and bypass have been employed for TICAs, but these approaches carry a high intraoperative rupture risk and are highly invasive [3]. With advances in endovascular therapy, coil embolization and stent-assisted coiling have been applied. However, the absence of a true aneurysm wall in pseudoaneurysms raises concerns regarding coil migration and rebleeding [4]. The advent of FDS has enabled reconstructive treatment that diverts flow away from the aneurysm while preserving the parent artery (Table 1). FDS promote vessel remodeling through endothelialization and are effective even in anatomically challenging locations or in lesions lacking a defined wall structure [15].

**Table 1:** Review of prior studies on standalone FDS placement for adult traumatic intracranial ICA pseudoaneurysm ASA, acetylsalicylic acid; CO, complete occlusion; DAPT, dual antiplatelet therapy; DSA, digital subtraction angiography; FDS, flow diverter stents; ICA, internal carotid artery; ICuPED, intraoperative complications unrelated to Pipeline Embolization Device; MPC, 2-methacryloyloxyethyl phosphorylcholine; No-FET, no further endovascular treatment required; PED, Pipeline Flex Embolization Device; TA, traffic accident; TICA, traumatic intracranial aneurysm

Study	Age (y)/sex	Etiology	TICA location	MPC polymer with PED	Complications	Pre-interventional antiplatelet regimen	Post-interventional antiplatelet regimen	Imaging follow-up (months)	Angiographic outcome
Kim et al. [5]	48/F	Head trauma	ICA-ophthalmic artery	None	None	NA	NA	6	CO
Sami et al. [6]	Middle-aged adult/NA	TA	Paraclinoid	None	ICuPED	ASA 325 mg + clopidogrel 75 mg for one week	DAPT for six months; ASA continued indefinitely	24	CO
Sami et al. [6]	Young adult/NA	TA	Cavernous	None	ICuPED	ASA 325 mg + clopidogrel 75 mg for one week	DAPT for six months; ASA continued indefinitely	3	CO
Sami et al. [6]	Young adult/NA	TA	Cavernous	None	ICuPED	ASA 325 mg + clopidogrel 75 mg for one week	DAPT for six months; ASA continued indefinitely	0	No follow-up DSAs
Deng and Feng [7]	22/M	Head trauma	Petrous-cavernous junction	None	None	ASA 100 mg + clopidogrel 75 mg for three days	NA	12	CO
Deng and Feng [7]	26/M	TA	Cavernous	None	None	ASA 100 mg + clopidogrel 75 mg for three days	NA	12	CO
Deng and Feng [7]	36/F	Head trauma	Petrous	None	None	ASA 100 mg + clopidogrel 75 mg for three days	NA	12	CO
Dogan et al. [8]	29/M	Head trauma	Cavernous	None	None	ASA 300 mg + clopidogrel 300 mg just before treatment	DAPT for six months; ASA continued indefinitely	7	No-FET
Dogan et al. [8]	20/M	Head trauma	Cavernous	None	None	ASA 300 mg + clopidogrel 300 mg just before treatment	DAPT for six months; ASA continued indefinitely	32	No-FET
Dogan et al. [8]	61/M	TA	Petrous	None	None	ASA 300 mg + clopidogrel 300 mg just before treatment	DAPT for six months; ASA continued indefinitely	16	No-FET
Dogan et al. [8]	30/M	TA	Cavernous	None	None	ASA 300 mg + clopidogrel 300 mg just before treatment	DAPT for six months; ASA continued indefinitely	21	No-FET
Dogan et al. [8]	57/F	Head trauma	Cavernous	None	None	ASA 300 mg + clopidogrel 300 mg just before treatment	DAPT for six months; ASA continued indefinitely	20	No-FET
Watanabe et al. [9]	20/F	TA	Cavernous	None	None	ASA 100 mg + prasugrel 3.75 mg for five days	NA	3	CO
Our case	46/F	TA	Petrous-cavernous junction	Present	None	ASA 300 mg just before treatment	ASA continued indefinitely	7	CO

In nontraumatic aneurysms treated with FDS, complete occlusion rates reach 77% at one year, 84.5% at two years, and 96% at five years [16]. However, their short-term efficacy in preventing rupture remains uncertain. Although systematic data on occlusion timing in TICAs are lacking, published series on FDS monotherapy report no delayed ruptures or need for retreatment, with most cases demonstrating progressive occlusion over time (Table 1). In our patient, angiography on day 17 confirmed complete occlusion, underscoring the short-term efficacy of FDS in this setting.

Because FDS have high metal density and thrombogenic potential, DAPT is generally required [15]. In polytrauma or post-craniectomy patients, however, DAPT carries a substantial bleeding risk. In the present case, characterized by severe brain contusion and recent decompressive craniectomy, we deemed DAPT too hazardous. To reduce thrombogenicity, we selected a third-generation PED-Shield, surface-modified with a 2-methacryloyloxyethyl phosphorylcholine (MPC) polymer. The MPC polymer offers high biocompatibility, allowing for uninhibited neointimal growth, and has been shown to effectively reduce thrombotic events clinically [17].

We chose aspirin as the sole antiplatelet agent for its well-established balance of efficacy and safety in neurosurgical settings. Although various antiplatelet agents are used globally, in Japanese clinical practice, aspirin and clopidogrel are most commonly considered for flow diverter placement. However, the antiplatelet effect of clopidogrel can be unpredictable in a significant portion of the Japanese population due to CYP2C19 gene polymorphisms, which may lead to insufficient antiplatelet response [18]. Given the high-risk, unpredictable nature of a polytrauma patient, our priority was to ensure a stable and reliable antiplatelet effect while minimizing hemorrhagic risk. Therefore, aspirin was selected for its consistent pharmacological profile, providing a more dependable single-agent therapy in this challenging clinical context.

Our strategy is supported by several reports demonstrating the feasibility of minimized antiplatelet therapy with PED-Shield in nontraumatic settings. For instance, Bounajem et al. [15] reported four cases of ruptured ICA pseudoaneurysms treated with PED-Shield under aspirin alone without thromboembolic complications. Similarly, Chiu et al. [19] described a ruptured dissecting vertebral aneurysm managed with minimized antiplatelet therapy. Recent efforts have also explored shortening DAPT duration or transitioning to SAPT after FDS deployment in nontraumatic cases, reflecting growing interest in reducing antiplatelet burden [20].

However, these prior reports primarily addressed nontraumatic pathologies, where the clinical context and hemorrhagic risks differ markedly from those in severe polytrauma patients. Our case represents a crucial advancement, as it is the first documented instance of successfully applying this SAPT strategy for a TICA, a lesion with inherently higher risks. This pioneering application demonstrates that the antithrombogenic properties of PED-Shield can extend beyond elective cases, offering a viable solution for highly challenging scenarios where DAPT is contraindicated. Guided by these prior reports and the unique characteristics of our device, we achieved a favorable outcome with PED-Shield and SAPT, suggesting that this approach can balance efficacy and safety in TICA management.

Nonetheless, the evidence supporting PED-Shield with SAPT remains limited to small series and case reports. Careful risk stratification and vigilant follow-up are essential, and treatment decisions should be individualized based on aneurysm location, morphology, and bleeding risk. Our case represents a pioneering application of aspirin-only antiplatelet management with PED-Shield for TICAs, and further clinical data are needed to refine antiplatelet strategies in the traumatic setting.

## Conclusions

This report describes the first documented case of a TICA successfully treated with FDS under SAPT without complications. FDS provides the dual advantages of parent vessel preservation and durable aneurysm exclusion. In polytrauma patients, a surface-modified FDS with antithrombogenic properties may mitigate the constraints associated with DAPT. This pioneering application of single-antiplatelet management highlights a significant advancement in TICA treatment, particularly for patients at high hemorrhagic risk. The strategy represents a valuable addition to the therapeutic armamentarium for TICAs and offers a promising approach for high-risk patients. Nevertheless, it emphasizes the urgent need for additional clinical data from larger cohorts and dedicated registries to refine antiplatelet strategies and validate this approach in the traumatic setting.
